# Selection of Suitable Reference Genes for RT-qPCR Analyses in Cyanobacteria

**DOI:** 10.1371/journal.pone.0034983

**Published:** 2012-04-04

**Authors:** Filipe Pinto, Catarina C. Pacheco, Daniela Ferreira, Pedro Moradas-Ferreira, Paula Tamagnini

**Affiliations:** 1 IBMC - Instituto de Biologia Molecular e Celular, Universidade do Porto, Porto, Portugal; 2 Departamento de Biologia, Faculdade de Ciências, Universidade do Porto, Porto, Portugal; 3 ICBAS - Instituto de Ciências Biomédicas Abel Salazar, Universidade do Porto, Porto, Portugal; University of New South Wales, Australia

## Abstract

Cyanobacteria are a group of photosynthetic prokaryotes that have a diverse morphology, minimal nutritional requirements and metabolic plasticity that has made them attractive organisms to use in biotechnological applications. The use of these organisms as cell factories requires the knowledge of their physiology and metabolism at a systems level. For the quantification of gene transcripts real-time quantitative polymerase chain reaction (RT-qPCR) is the standard technique. However, to obtain reliable RT-qPCR results the use and validation of reference genes is mandatory. Towards this goal we have selected and analyzed twelve candidate reference genes from three morphologically distinct cyanobacteria grown under routinely used laboratory conditions. The six genes exhibiting less variation in each organism were evaluated in terms of their expression stability using geNorm, NormFinder and BestKeeper. In addition, the minimum number of reference genes required for normalization was determined. Based on the three algorithms, we provide a list of genes for cyanobacterial RT-qPCR data normalization. To our knowledge, this is the first work on the validation of reference genes for cyanobacteria constituting a valuable starting point for future works.

## Introduction

Cyanobacteria are a multifaceted group of photosynthetic prokaryotes, with a large morphological diversity including unicellular, colonial and filamentous forms. Some filamentous strains exhibit cellular differentiation, with cells specialized in photosynthesis (vegetative cells) and in nitrogen fixation (heterocysts) [1]–[2]. Besides the morphological diversity, cyanobacteria are versatile microorganisms with simple growth requirements that are able to produce several secondary metabolites of commercial interest [3]–[5]. To exploit this biotechnological potential and use cyanobacteria as cell factories it is necessary to understand their physiology and metabolism at a systems level. The comprehensive evaluation of gene expression patterns is an important part of this characterization and, at present, the quantitative real-time polymerase chain reaction (RT-qPCR) is considered the gold standard for measurement of transcript abundance [6]. This technique allows the simultaneous amplification and quantification of a target amplicon by measuring the fluorescence increment in each PCR cycle in a fast, extremely sensitive and accurate way [7]. However, it has been shown that the poor design of RT-qPCR experiments can lead to erroneous or biologically irrelevant data [6]. A number of parameters such as the sample origin and amount, RNA quality and integrity, PCR efficiency, qPCR protocol and validation have been shown to compromise the quality of the final RT-qPCR experiment [8]–[10]. Additionally, the data normalization strategy used to eliminate technical or experimentally induced variation is very important. Normalization against internal control genes is frequently used, yet the expression of housekeeping genes can vary significantly depending on the samples and experimental conditions [11]–[14]. To minimize the influence of individual variation experimental validation of each reference gene is required for a particular sample and condition, and the use of three or more reference genes is now standard practice [10], [15]. Moreover, several algorithms have been developed to allow the evaluation of candidate reference genes in terms of their expression stability and determination of the minimum number of reference genes to be used [16]–[19].

In this work we have validated candidate reference genes for accurate normalization of RT-qPCR data in cyanobacteria. Several genes were selected and their transcription analyzed in three morphological distinct organisms: the unicellular *Synechocystis* sp. PCC 6803 (*Synechocystis*), the filamentous *Lyngbya aestuarii* CCY 9616 (*Lyngbya*), and the filamentous heterocystous *Nostoc* sp. PCC 7120 (*Nostoc*). Our analysis provides a list of reference genes that can be used in RT-qPCR experiments with these cyanobacteria, grown in conditions routinely used in the laboratory. Although validation is always mandatory, our work constitutes a valuable starting point for the selection of reference genes, even for other cyanobacterial strains.

## Results and Discussion

### Choice of candidate reference genes

Two approaches were followed to choose the candidate reference genes to be validated: (*i*) genes that have been used as controls in previous RT-qPCR studies and (*ii*) genes whose transcription did not vary significantly in two microarray studies [20]–[21] available in the CyanoBIKE server [22]. Twelve genes from independent pathways were selected to minimize the effects of co-regulation (Table 1): seven based on the bibliography (*i*) and five from CyanoBIKE (*ii*). A preliminary RT-qPCR analysis was performed using cells grown under a 16 h light/8 h dark regimen (LD) and collected in the middle of the phases (L8 and D4), in medium with combined nitrogen (N^+^) or without combined nitrogen (N^−^) for *Lyngbya* and *Nostoc*, while in *Synechocystis* only N^+^ medium was used (non-N_2_-fixing strain). The six candidate reference genes with lowest Cq variation were selected for further studies in each organism: *rrn16S* (16S), *rnpB* and *secA* (tested in all organisms), *ilvD* (*Nostoc*), *petB* (*Nostoc* and *Synechocystis*), *ppc* (*Lyngbya* and *Synechocystis*), *purC* (*Lyngbya*), *rnpA* (*Lyngbya* and *Nostoc*) and *rpoA* (*Synechocystis*). In the second RT-qPCR analysis, samples collected in continuous light (CL) were included and four time points were tested in all conditions (CL.N^+^, CL.N^−^, LD.N^+^ and LD.N^−^).

**Table 1:** Candidate reference genes evaluated in this study.

Approach	Gene	Description	Reference
**Bibliographic search**	*rrn16S*	16S ribosomal RNA	[20], [31]–[37]
	*petB*	Cytochrome *b*_6_, involved in electron transport and ATP generation	[38]
	*ppc*	Phospho*enol*pyruvate carboxylase (PEPC), central enzyme in carbon concentrating mechanism	[39]
	*rnpA*	Protein subunit of ribonuclease P (RNase P)	[38]
	*rnpB*	RNA subunit of ribonuclease P	[40]–[44]
	*rps1B*	Small subunit ribosomal protein S1	[42], [44]
	*rpoA*	RNA polymerase, alpha subunit	[45]–[46]
**CyanoBIKE** *	*ilvD*	Dihydroxyacid dehydratase, participates in the biosynthetic process of branched chain amino acid family	This study
	*mrp*	Multiple drug resistance protein (MRP) homolog	This study
	*prsA*	Ribose-phosphate pyrophosphokinase, involved in the pentose phosphate pathway	This study
	*purC*	SAICAR synthetase, involved in the *de novo* purine biosynthetic pathway	This study
	*secA*	Part of the Sec protein translocase complex	This study

* Search based on the works by Ehira and Ohmori [20] and Hihara *et al.* [21].

### Amplicon specificity, RT-qPCR efficiencies and analysis of Cq values

The size of the amplicons was confirmed by agarose gel electrophoresis (one sample per primer pair per organism, see Fig. S1) and their specificity confirmed by DNA sequencing. In all the RT-qPCR runs, standard curves were included (using cDNA serial dilutions) and the amplification efficiencies (E) obtained were in the recommended range (90–110%), except for the *rpoA* gene (*Synechocystis*) in which E was slightly higher (117.4%, see Table 2). The candidate reference genes exhibited desirable E values with acceptable *R^2^* values demonstrating linearity and reproducibility of the reactions across organisms and variety of conditions tested. The melting curves showed single peaks corresponding to unique amplicons, and in the no template controls (NTCs) no amplification or non-specific products (unstable at the specific amplicon melting temperature) were detected (Table 2). The parameters derived from the RT-qPCR analysis required by the MIQE guidelines are listed in Table 2.

**Table 2:** Amplicon sizes and parameters derived from RT-qPCR data analysis.

Organism	Genes	Amplicon size (bp)	Amplicon T_m_ (°C)	Median Cq	NTC* (Cq)	Amplification efficiency	*R^2^*	Slope	y interception
***Lyngbya aestuarii*** ** CCY 9616**	*rrn16Sa.b*	191/192	86.50	21.035	31.12	101.3	0.972	−3.292	17.859
	*ppc*	90	82.00	30.93	43.51**	100.4	0.968	−3.313	29.319
	*purC*	112	80.00	32.72	35.30**	103.4	0.921	−3.243	29.339
	*rnpA*	133	81.50	30.895	35.34**	105.3	0.990	−3.201	28.23
	*rnpB*	189	86.00	32.79	N.d.	101.3	0.987	−3.291	29.104
	*secA*	131	83.00	31.92	53.68**	98.6	0.912	−3.355	26.701
***Nostoc*** ** sp. PCC 7120**	*rrn16Sa.b.c.d*	191	83.50	17.865	31.83	105.4	0.981	−3.198	16.516
	*ilvD*	77	79.50	29.21	N.d.	99.7	0.978	−3.329	25.359
	*petB*	157	82.50	30.235	N.d.	94.3	0.988	−3.466	30.284
	*rnpA*	204	82.50	33.37	37.69**	97.5	0.980	−3.383	30.194
	*rnpB*	131	84.00	23.9	N.d.	100.3	0.994	−3.314	20.060
	*secA*	131	81.50	30.785	N.d.	99.5	0.988	−3.333	28.202
***Synechocystis*** ** sp. PCC 6803**	*rrn16Sa.b*	190	86.00	18.425	28.84	100.4	0.981	−3.312	14.654
	*ppc*	108	83.50	34.4358	47.57**	95.9	0.960	−3.425	30.680
	*petB*	179	84.50	29.63	N.d.	101.5	0.958	−3.287	28.052
	*rnpB*	136	81.50	26.38	42.43**	103.8	0.982	−3.233	22.366
	*rpoA*	198	84.00	32.875	41.41**	117.4	0.991	−2.965	28.836
	*secA*	113	82.00	35.04	N.d.	102.1	0.958	−3.271	31.765

* No template control.

** Non-specific amplicons (unstable at the specific amplicon melting temperature).

N.d. – Not detected.

The raw quantification cycle (Cq) values were extracted from the iQ5 Optical System Software v2.1 (Bio-Rad) and represented by box-and-whiskers plots (Fig. S2). The median Cq value for the 16S was the lowest in all organisms (Cq≈20), which was expected since it is one of the most abundant transcripts. For the other genes, similar median Cq values were observed in *Lyngbya* (Cq≈30), while for *Nostoc* and *Synechocystis* larger variation was found (23<Cq≤36). The use of 16S as reference gene is controversial since the degradation machinery may not affect rRNA and mRNA in the same manner [23]–[24], and it is also recommended that reference and target genes should have similar Cq values [25]. The latter issue can be circumvented by ensuring that the calculations are performed within the linear range of the amplification curves of reference and target genes [25].

### Assessing gene-stability and minimum number of reference genes required

**Figure 1:**
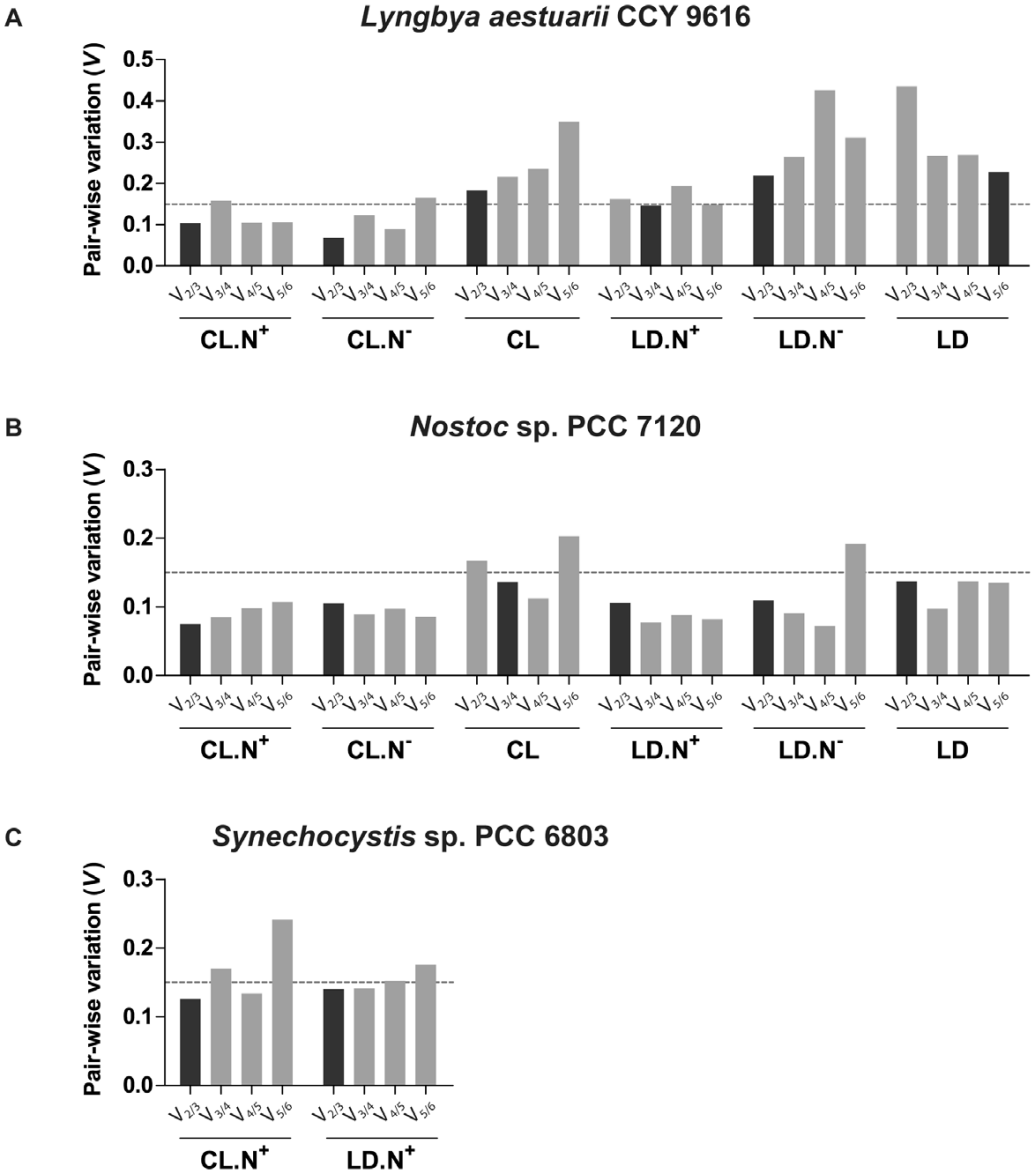
Pair-wise variation *V_n_*/*V_n_*_+1_ calculated by geNorm^PLUS^ for *Lyngbya* (A), *Nostoc* (B) and *Synechocystis* (C). Dark-grey bars indicate the minimum number of reference genes required for accurate normalization.

Gene expression stability was analyzed using three distinct algorithms: geNorm [16], NormFinder [18] and BestKeeper [17]. These algorithms rank the candidate reference genes according to the calculated gene expression stability values (*M*, geNorm and NormFinder) or Pearson's correlation coefficients (*r*, BestKeeper) (see Material and Methods for further details). Two types of analyses were performed to assess the stability: (*i*) under each growth condition (CL.N^+^, CL.N^−^, LD.N^+^ and LD.N^−^) pooling the data corresponding to four collection time points, or (*ii*) under each light regimen (CL and LD) pooling the data corresponding to eight collection time points (four in each medium). For each time point, data from each biological and technical replicates were considered. The analysis of reference genes stability using the different algorithms revealed similar gene rankings for all organisms under the different conditions (Tables S1, S2 and S3). Furthermore, the optimal number of reference genes for each organism in each specific experimental condition was determined using geNorm^PLUS^, which calculates the pair-wise variation – *V_n_*/*V_n_*_+1_. This analysis showed that, in most of the conditions, the value of *V*_2/3_ was below 0.15 indicating that the minimum number of reference genes required for normalization is two (Fig. 1). For *Lyngbya*, under the conditions CL, LD.N^−^ and LD the pair-wise variation was always above the threshold and therefore the use of three genes for the first two conditions and five for latter is necessary (Fig. 1A). Taking into consideration the ranking of the three algorithms as well as the pair-wise analysis of geNorm^PLUS^, a comprehensive selection of adequate reference genes is presented in Table 3. In all the conditions tested and in the three organisms, a minimum of three stable reference genes was always considered since it is more appropriate for a reliable normalization of data [16]. In this study the 16S gene was shown to be a stable reference gene in all organisms, and can be used for normalization in all the conditions tested in *Nostoc* (six conditions) and in *Synechocystis* (two conditions). The *rnpB* gene was stable in the three cyanobacteria, but the conditions in which it can be used are more limited. This first study on the validation of cyanobacterial reference genes identified 16S and *rnpB* as the most stable in the majority of the conditions tested; by coincidence these two genes were the more commonly used for normalization in previous cyanobacterial RT-qPCR studies (Table 1). The *rnpA* and *secA* genes can also be considered for data normalization in *Lyngbya* and *Nostoc* in a wide range of conditions. All the other reference genes listed in Table 3 are more strain-dependent: *ilvD* can be used in *Nostoc*, while *ppc* and *purC* are specific for *Lyngbya*, and *petB* is suggested for *Synechocystis* and *Nostoc*, but in the latter only in one condition (LD.N^−^).

**Table 3:** Recommended reference genes for *Lyngbya*, *Nostoc* and *Synechocystis*, under the conditions tested.

Organism	Condition*	Candidate reference genes**								
		16S	*ilvD*	*petB*	*ppc*	*purC*	*rnpA*	*rnpB*	*rpoA*	*secA*
***Lyngbya aestuarii*** ** CCY 9616**	CL.N**^+^**	−	ns	ns	**+**	**+**	−	−	ns	**+**
	CL.N^−^	−	ns	ns	**+**	−	**+**	−	ns	**+**
	CL***	−	ns	ns	−	**+**	**+**	−	ns	**+**
	LD.N**^+^**	**+**	ns	ns	**+**	−	**+**	−	ns	**+**
	LD.N^−^	**+**	ns	ns	−	**+**	**+**	−	ns	−
	LD***	−	ns	ns	**+**	**+**	**+**	**+**	ns	**+**
***Nostoc*** ** sp. PCC 7120**	CL.N**^+^**	**+**	−	−	ns	ns	**+**	−	ns	**+**
	CL.N^−^	**+**	−	−	ns	ns	**+**	**+**	ns	−
	CL***	**+**	**+**	−	ns	ns	−	**+**	ns	**+**
	LD.N**^+^**	**+**	−	−	ns	ns	**+**	−	ns	**+**
	LD.N^−^	**+**	**+**	**+**	ns	ns	−	−	ns	**+**
	LD***	**+**	−	−	ns	ns	**+**	−	ns	**+**
***Synechocystis*** ** sp. PCC 6803**	CL.N**^+^**	**+**	ns	**+**	−	ns	ns	**+**	−	−
	LD.N**^+^**	**+**	ns	**+**	−	ns	ns	**+**	−	−

* CL – continuous light; LD – light/dark regimen; N^+^ – medium with combined nitrogen; N^−^ – medium without combined nitrogen.

** Genes recommended (+), not recommended (−) or not selected after preliminary analysis (ns).

*** Calculation performed pooling data from cells grown in both media and in the same light regimen.

### Choice of reference genes

**Figure 2:**
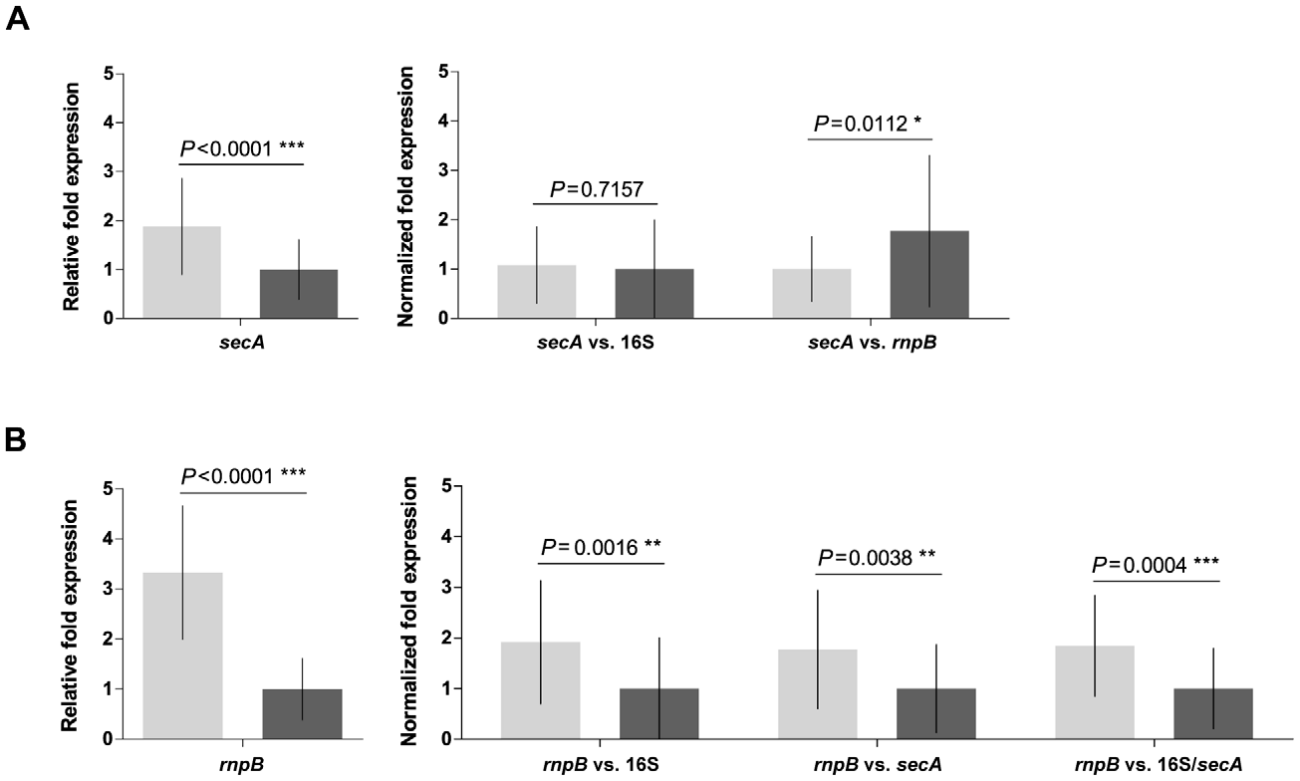
Effect of choice of reference gene(s). Relative (left) and normalized (right) fold expression of *secA* (A) and *rnpB* (B). Expression normalization was performed using data from the three biological replicates of *Nostoc* grown under a light/dark regimen, in N^+^ (light-grey) or N^−^ (dark-grey) medium, using different genes as calibrators. Error bars indicate one standard error of the mean.

To demonstrate the effects of the choice of a non-optimal reference gene, an expression analysis was simulated using data from *Nostoc* grown under a light/dark regimen. In this particular condition, 16S was identified as the most stable gene, followed by *secA*, and *rnpB* was identified as the least stable. Therefore, the simulation was performed considering *secA* as target and its expression was normalized against 16S or *rnpB* (Fig. 2A). The relative fold expression of *secA* was calculated and normalized to the lowest value: 1.88±0.98 in N^+^ and 1.00±0.61 in N^−^ medium. This normalization resulted in a significant difference in relative fold expression when comparing the two media (*P*<0.0001). The expression level of *secA* was calculated relative to 16S, and it was found to be stable in both media, *P* = 0.7157 (1.08±0.77 in N^+^ medium and 1.00±1.00 in N^−^ media). In contrast, when *rnpB* was used as reference the relative expression of *secA* varied between 1.00±0.66 and 1.77±1.53 in N^+^ and N^−^ media, respectively, with a significant *P* value of 0.0112. Consequently, the difference between gene expression levels of *secA* is an artifact caused by the wrong choice of reference gene. This finding is supported by the results of the gene expression stability for *secA* (see above). A second analysis was performed taking *rnpB* as target and 16S and *secA* as reference genes (Fig. 2B). The *rnpB* relative fold expression was also calculated and normalized to the lowest value: 3.32±1.33 in N^+^ and 1.00±0.61 and N^−^ medium (*P*<0.0001). Subsequently, *rnpB* expression was normalized to 16S, *secA* or both genes generating similar results: *rnpB* expression was always significantly lower in N^−^ medium. Overall, this analysis confirms the expression stability of 16S and *secA* genes and the inaccuracy of using the *rnpB* gene as a reference, emphasizing that the choice of non-optimal reference genes leads to data misinterpretation.

### Conclusions

This is the first study on validation of reference genes from cyanobacteria and, although a “universal” set could not be identified, a list of recommended genes is provided for the normalization of RT-qPCR data. The expression of a set of candidate reference genes was analyzed in three morphologically distinct cyanobacteria grown under different conditions. The different algorithms used to assess expression stability did not rank the candidate genes in the same order, but in general identified the same as the most stable. This reinforces not only the need to use more than one algorithm for accurate validation, but also the requirement of normalizing data against three or more reference genes. The array of experimental conditions tested makes this work a valuable starting point in the choice of reference genes when studying cyanobacteria.

## Materials and Methods

### Strains, culture conditions and sample collection

Three cyanobacterial strains, representing different sections among cyanobacteria, were used: the unicellular *Synechocystis* sp. PCC 6803 (Pasteur Culture Collection of Cyanobacteria, Paris, France), the filamentous non-heterocystous *Lyngbya aestuarii* CCY 9616 (Culture Collection Yerseke, NIOO-KNAW, The Netherlands; co-identity *Lyngbya* sp. PCC 8106), and the filamentous heterocystous *Nostoc* sp. PCC 7120 (Pasteur Culture Collection of Cyanobacteria, Paris, France). *Lyngbya* was grown in artificial seawater ASN-III or ASN_0_-III (ASN-III devoid of nitrate) [26], *Nostoc* was grown in BG11 or BG11_0_ and *Synechocystis* only in BG11 [27]. The cyanobacterial cultures were kept at 25°C with agitation, on a continuous light (20 µmol photons m^−2^ s^−1^, CL) or 16 h light (15 µmol photons m^−2^ s^−1^)/8 h dark (LD) regimen. For sample collection, three independent cultures of the organisms were grown for three to four days (under the different media and light conditions), at this stage the cells were changed to new medium and sample collection started after 24 h. For cultures in light/dark regimen, samples were collected in the transitions between the dark and the light phases (L0), in the middle of the light phase (eight hours into the light phase, L8), in the transition between the light and the dark phases (D0) and in the middle of the dark phase (four hours into the dark phase, D4). For continuous light, the first sample was collect 24 h after medium change (L0), and the remaining samples were collected after 8 h (L8), 16 h (L16) and 20 h (L20), time-points equivalent to the light/dark regimen sample collection. The mass or volumes of samples collected were 0.1–0.3 g for *Lyngbya*, 20 mL for *Nostoc* and 40 mL for *Synechocystis*. Cells were spun down (8 min at 4500 *g*, 4°C), resuspended in 0.5 mL medium and mixed with 1 mL RNAprotect® Bacterial reagent (Qiagen) and processed according to manufacturer's instructions, the cell pellets were frozen at −80°C until further use.

*Escherichia coli* strain DH5α (Stratagene) was used for cloning purposes. *E. coli* transformants were cultivated at 37°C in Luria-Bertani (LB) medium supplemented with 100 µg mL^−1^ ampicillin.

### Candidate reference genes, primer design and amplicon specificity

The selection of the candidate reference genes was performed by bibliographic search, and using the CyanoBIKE server (http://biobike.csbc.vcu.edu:8003, accessed 2008) [22] selecting genes whose relative transcript levels varied between 0.7 and 1.3 but their difference in all conditions tested was below 0.5.

The sequences of the twelve selected genes were obtained from GenBank (accession number and location in Table S4). The primers were designed using the Beacon Designer 6 software (PREMIER Biosoft International) and synthesized (with cartridge purification) by STAB Vida (Lisbon). For each primer pair, the annealing temperature was optimized through gradient PCR, in reaction mixtures (20 µL) containing: 0.25 µM of each primer, 10 µL of iQ™ SYBR® Green Supermix (Bio-Rad) and 2 µL cDNA (1/9 dilution). The PCR profile was: 3 min at 95°C followed by 60 cycles of 30 s at 95°C, 30 s at 51, 53 or 56°C (Table 4 and Table S5) and 30 s at 72°C. A melting curve analysis was performed at the end of each PCR program, to exclude the formation of nonspecific products. After optimization, the size of the PCR products (for each gene and in each organism) was checked by agarose gel electrophoresis performed by standard protocols, using 1× TAE buffer [28]. These products were also cloned into the pGEM-T® Easy (Promega) vector according to the manufacturer's instructions, and the amplicon specificity was confirmed by DNA sequencing (STAB Vida).

**Table 4:** Primer nucleotide sequences and annealing temperatures (T_a_) used in RT-qPCR.

Organism	Genes	Primer name	Primer Sequence (5′→3′)	T_a_ (°C)
***Lyngbya aestuarii*** ** CCY 9616**	*rrn16Sa.b*	BD16SF1	CACACTGGGACTGAGACAC	56
		BD16SR1	CTGCTGGCACGGAGTTAG	
	*ppc*	ppcF	TATCGCCAAGAACCCTATCG	56
		LppcR	GCTATTGTAAAGTCGCCAG	
	*purC*	purCF	GATACCTGTCGTTTGTGG	56
		LpurCR	GAACTTGTTGATAAGCCG	
	*rnpA*	rnpAF1	AACGTCAAATTCGAGCCG	56
		rnpAR1	AACAACTGCTCTAATTCTTGC	
	*rnpB*	LrnpBF	ATCATCTGTTGTTGGTAAGG	51
		LrnpBR	GAGGGCAATTATCTATCTGG	
	*secA*	secAF	AACCTACTACTACGACATCC	51
		secAR	ACTTAATCACCTGTTCTTTCAA	
***Nostoc*** ** sp. PCC 7120**	*rrn16Sa.b.c.d*	BD16SF1	CACACTGGGACTGAGACAC	51
		BD16SR1	CTGCTGGCACGGAGTTAG	
	*ilvD*	ilvDF	GGCTGTGATAAGAATATGC	56
		ilvDR	CCACCGTAAACAAAGATAG	
	*petB*	petBF1	AGTTTGCCACTGGATTCG	56
		petBR1	AGAATCATCATCAACACCATC	
	*rnpA*	NrnpAF	TACGCTCATTGGTGTCTCG	56
		rnpAR1	AACAACTGCTCTAATTCTTGC	
	*rnpB*	NrnpBF2	TAGGGAGAGAGTAGGCGTTG	56
		NrnpBR2	TTCTGTGGCACTATCCTCAC	
	*secA*	secAF	AACCTACTACTACGACATCC	56
		secAR	ACTTAATCACCTGTTCTTTCAA	
***Synechocystis*** ** sp. PCC 6803**	*rrn16Sa.b*	BD16SF1	CACACTGGGACTGAGACAC	51
		BD16SR1	CTGCTGGCACGGAGTTAG	
	*ppc*	ppcF	TATCGCCAAGAACCCTATCG	56
		SppcR	CATCGTTTGCCGTTCTTCTG	
	*petB*	SpetB1F	CCTTCGCCTCTGTCCAATAC	56
		SpetB1R	TAGCATTACACCCACAACCC	
	*rnpB*	rnpBF1	CGTTAGGATAGTGCCACAG	56
		rnpBR1	CGCTCTTACCGCACCTTTG	
	*rpoA*	SrpoAF	TTGGACTAATGGCAGTATTCA	56
		rpoAR1	CGCTTGAGACAGTTATAGGC	
	*secA*	secAF	AACCTACTACTACGACATCC	51
		SsecAR	TTAAATCCAAACCTTCCAG	

### RNA extraction and cDNA synthesis

For RNA extraction, the TRIzol® Reagent (Ambion) was used in combination with the Purelink™ RNA Mini Kit (Ambion). Briefly, the cells were disrupted in TRIzol containing 0.2 g of 0.2 mm-diameter glass beads (acid washed, Sigma) using a Mini-Beadbeater (Biospec Products) or a FastPrep®-24 (MP Biomedicals) and the following extraction steps were performed according to the manufacturer's instructions. RNA was quantified on a NanoDrop ND-1000 spectrophotometer (NanoDrop Technologies, Inc.) and the quality and integrity was checked using the Experion™ RNA StdSens Analysis Kit (Bio-Rad). The RNA samples (≈300 ng) were treated with 1 U of RQ1 RNase-free DNase (Promega) according to manufacturer's instructions. The absence of genomic DNA contamination was checked by PCR, in reaction mixtures containing: 0.5 U of GoTaq Flexi DNA Polymerase (Promega), 1× GoTaq Flexi buffer, 200 µM of each deoxyribonucleotide triphosphate (dNTP), 1 µM of each BD16S primer (Table 4), and 2 µL total RNA. The PCR profile was: 2 min at 95°C followed by 25 cycles of 30 s at 95°C, 30 s at 51°C and 30 s at 72°C, and a final extension at 72°C for 7 min. The PCR reactions were run on agarose gel electrophoresis.

For cDNA synthesis, 150 ng of total RNA was transcribed with the iScript™ Reverse Transcription Supermix for RT-qPCR (Bio-Rad) in a final volume of 20 µL, following the manufacturer's instructions. A control PCR was performed using 1 µL of cDNA as template and the same reaction conditions and PCR program described above. Three-fold standard dilutions of the cDNAs were made (1/3, 1/9 and 1/27) and stored at −20°C.

### Transcription analysis by Real-time RT-PCR

The RT-qPCRs were performed on iQ™ 96-well PCR plates covered with Optical Sealing Tape (Bio-Rad). Reactions were manually assembled and contained 0.25 µM of each primer, 10 µL of iQ™ SYBR® Green Supermix (Bio-Rad) and 2 µL of template cDNA (dilution 1/9). The PCR profile was: 3 min at 95°C followed by 60 cycles of 30 s at 95°C, 30 s at 51 or 56°C (Table 4) and 30 s at 72°C. Standard dilutions of the cDNA were used to check the relative efficiency and quality of primers. Negative controls (no template cDNA) were included and a melting curve analysis was performed in all assays. RT-qPCRs were performed with three biological replicates and technical triplicates/duplicates of each cDNA sample in the iCycler iQ5 Real-Time PCR Detection System (Bio-Rad). The data obtained were analyzed using the iQ5 Optical System Software v2.1 (Bio-Rad). Efficiency values were calculated and the Cq values for each data set were exported to a Microsoft Office Excel file, and imported into the qbase^PLUS2^ software (Biogazelle). The relative quantities of each sample were calculated using the gene-specific efficiency acquired from the dilutions series and normalized to the mean Cq value.

GraphPad Prism v5 (GraphPad Software, Inc.) was used to create the box-and-whisker plots. For each gene in a given condition, the Cq values of all technical replicates corresponding to the four collection time points were pooled together. Boxes correspond to Cq values within the 25^th^ and 75^th^ percentiles and the median is represented by an horizontal line. Whiskers include Cq values within the 10^th^ and the 90^th^ percentiles and Cq values outside this range (outliers) are represented as dots (Fig. S2).

### Analyses of gene stability and number of optimal reference genes

Gene stability was assessed using three different mathematical algorithms: geNorm^PLUS^ [16], NormFinder [18], and BestKeeper [17]. The geNorm^PLUS^ algorithm determines internal control gene stability measure (*M*) as the average pair-wise variation of each reference gene with all the other reference genes, and enables the elimination of the least stable gene and the recalculation of the *M* values, resulting in the ranking of the most stable genes. The lower the *M* value, the higher is the gene stability; a good reference gene should have an *M* below 0.5 in homogeneous sample sets [15], [29]. Furthermore, the minimum number of genes required for the calculation of a reliable normalization factor is determined, calculating the pair-wise variation *V_n_*/*V_n_*_+1_ between two sequential normalization factors *NF_n_* and *NF_n_*_+1_ (geometric mean of the expression levels of *n* reference genes). The stepwise inclusion of reference genes is performed until *V_n_*/*V_n_*_+1_ drops below the recommended threshold of 0.15, when the benefit of using an extra gene (*n*+1) is limited [16], [30]. Gene stability determination by geNorm^PLUS^ was performed using the qbase^PLUS2^ software v2.2 (Biogazelle).

NormFinder focuses on the expression variations both between different groups (inter-group variation) and inside one group (intra-group variation), in a model-based approach of mixed linear effect modeling. This algorithm determines inter- and intra-group variations and combines both results in a stability value for each investigated gene. According to NormFinder, genes with lowest *M* value will be top ranked. For the analysis with this algorithm, expression values were calculated using qbase^PLUS2^ and first exported to a Microsoft Office Excel file for use with the NormFinder applet.

BestKeeper calculates standard deviations (*SD*) and the coefficient of variance (*CV*) based on the Cq values of all candidate reference genes, considering the genes with *SD* lower than 1 as stably expressed. The algorithm calculates the BestKeeper index (*BI*) as the geometric mean of the stable reference genes Cq values and then establishes pair-wise correlations between each gene and the *BI*. The genes with highest Pearson correlation coefficient (*r*≈1) and probability (*p*) value lower than 0.05 are considered the most stable. The average Cq value of each technical triplicate/duplicate was calculated (without conversion to relative quantity) and imported to the Microsoft Excel based application BestKeeper to analyze gene stability.

### Choice of reference genes

The gene expression simulation was performed using data from the three biological replicates of *Nostoc* grown under a light/dark regimen, pooling the data from the different time collection points (*n* = 32). Relative and normalized fold expression values were calculated using the iQ5 Optical System Software v2.1 (Bio-Rad). Expression data were imported to GraphPad Prism v5 (GraphPad Software, Inc.) and the statistical significance of the results was assessed through the Student's *t* test.

### MIQE guidelines

In this work, the Minimum Information for Publication of Quantitative Real-Time PCR Experiments (MIQE) guidelines [10] were followed to promote the effort for experimental consistency and transparency, and to increase the reliability and integrity of the results obtained.

## Supporting Information

Figure S1 **Confirmation of amplicon sizes for the selected candidate reference genes studied in each cyanobacteria.** Agarose gel electrophoresis showing specific PCR products of the expected sizes for each candidate reference gene in *Lyngbya* (A), *Nostoc* (B) and *Synechocystis* (C). MM – GeneRuler™ 50 bp DNA Ladder (Fermentas).
(TIF)

Figure S2 Box-and-whiskers plots of candidate reference gene Cq values in *Lyngbya* (A), *Nostoc* (B) and *Synechocystis* (C). Boxes correspond to Cq values within the 25^th^ and 75^th^ percentiles and the median is represented by an horizontal line. Whiskers include Cq values within the 10^th^ and the 90^th^ percentiles and Cq values outside this range (outliers) are represented as dots.
(TIF)

Table S1 Ranking of the candidate reference genes according to their stability value (*M*) calculated by geNorm.
(DOC)

Table S2 Ranking of the candidate reference genes according to their stability value (*M*) calculated by NormFinder.
(DOC)

Table S3 Ranking of the candidate reference genes according to the Pearson's correlation coefficient (*r*) calculated against the BestKeeper index and probability values (*p*).
(DOC)

Table S4 Candidate reference genes accession number and location.
(DOC)

Table S5 Primer nucleotide sequences of genes tested only in preliminary RT-qPCR analysis.
(DOC)
