# Supplementary figures and images for: Selection of Suitable Reference Genes for RT-qPCR Analyses in Cyanobacteria
Source: PLoS One. 2012 Apr 4;7(4):e34983. doi: 10.1371/journal.pone.0034983 (PMC3319621; doi:10.1371/journal.pone.0034983)

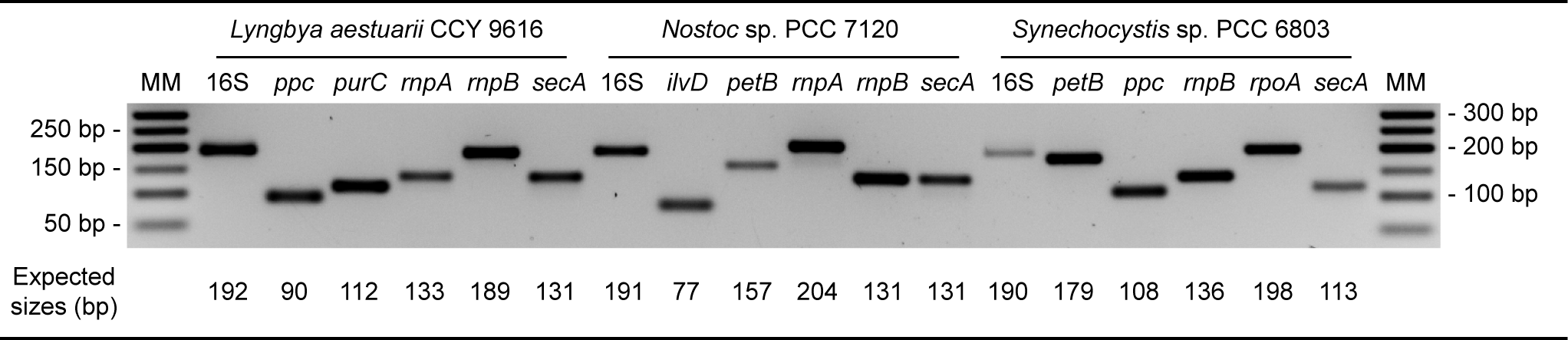

Supplement: Figure S1 — Confirmation of amplicon sizes for the selected candidate reference genes studied in each cyanobacteria. Agarose gel electrophoresis showing specific PCR products of the expected sizes for each candidate reference gene in Lyngbya (A), Nostoc (B) and Synechocystis (C). MM – GeneRuler™ 50 bp DNA Ladder (Fermentas). (TIF) [file pone.0034983.s001.tif]

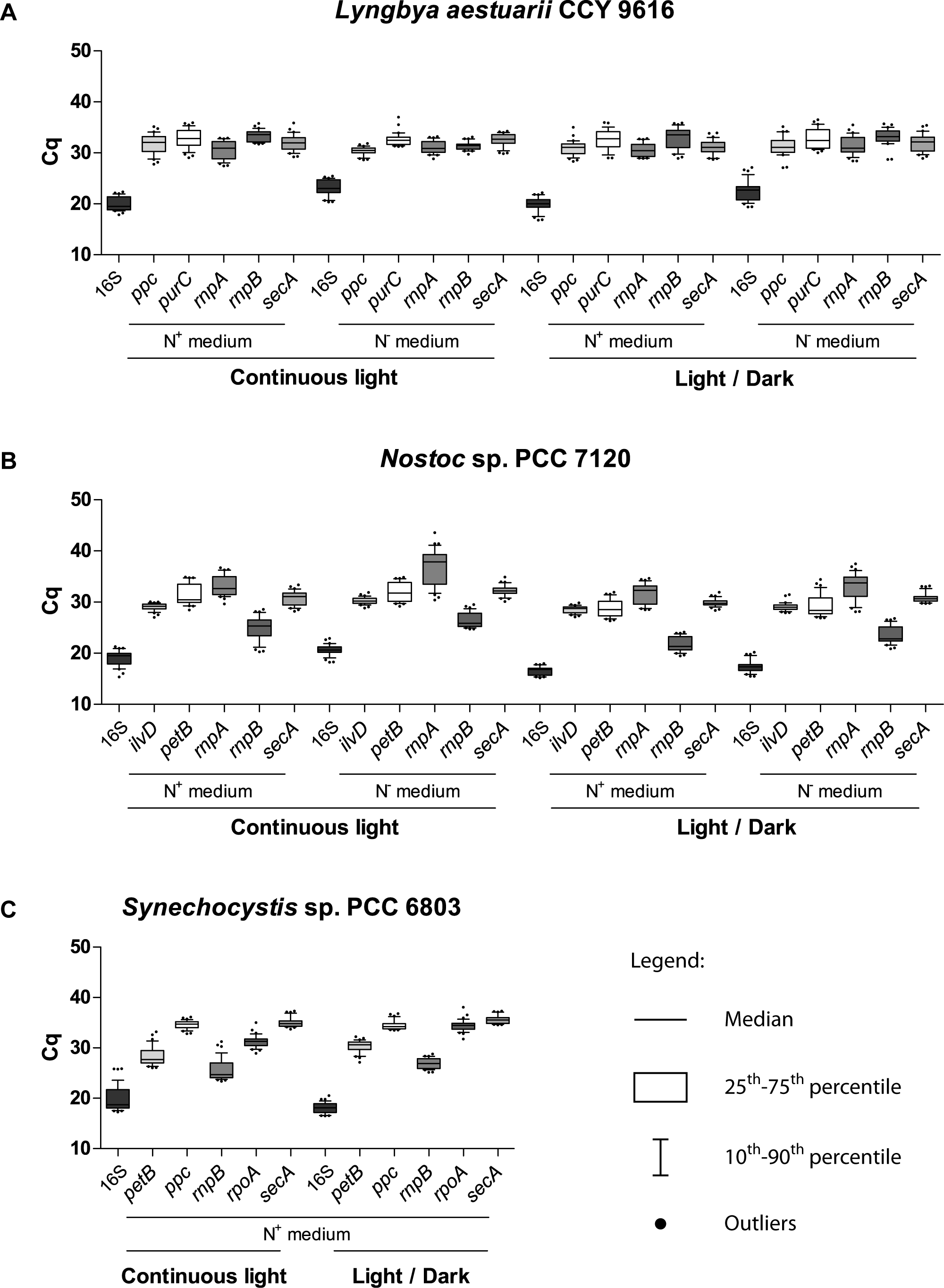

Supplement: Figure S2 — Box-and-whiskers plots of candidate reference gene Cq values in Lyngbya (A), Nostoc (B) and Synechocystis (C). Boxes correspond to Cq values within the 25th and 75th percentiles and the median is represented by an horizontal line. Whiskers include Cq values within the 10th and the 90th percentiles and Cq values outside this range (outliers) are represented as dots. (TIF) [file pone.0034983.s002.tif]
