# Supplementary material for: Selection of Suitable Reference Genes for RT-qPCR Analyses in Cyanobacteria
Source: PLoS One. 2012 Apr 4;7(4):e34983. doi: 10.1371/journal.pone.0034983 (PMC3319621; doi:10.1371/journal.pone.0034983)
Supplement: Table S1 — Ranking of the candidate reference genes according to their stability value (M) calculated by geNorm. (DOC) [file pone.0034983.s003.doc]

**Table S1.** Ranking of the candidate reference genes according to their stability value (M) calculated by geNorm.

| **Organism** | **Condition^*^** |  | **Ranking (less stable to more stable)^**^** | | | | | |
| --- | --- | --- | --- | --- | --- | --- | --- | --- |
|  |  |  | 6 | 5 | 4 | 3 | 2 | 1 |
| ***Lyngbya aestuarii* CCY 9616** | CL.N^+^ |  | *rnpB* | *rnpA* | 16S | *purC* | ***ppc*** | ***secA*** |
|  |  | *M* | 0.572 | 0.512 | **0.453** | **0.260** | **0.231** | **0.208** |
|  | CL.N^-^ |  | 16S | *rnpB* | *purC* | *rnpA* | ***secA*** | ***ppc*** |
|  |  | *M* | 0.609 | **0.405** | **0.337** | **0.171** | **0.153** | **0.137** |
|  | CL^***^ |  | 16S | *rnpB* | *ppc* | ***purC*** | ***rnpA*** | ***secA*** |
|  |  | *M* | 1.329 | 0.929 | 0.728 | 0.545 | 0.521 | 0.505 |
|  | LD.N^+^ |  | *rnpB* | *purC* | *ppc* | **16S** | ***rnpA*** | ***secA*** |
|  |  | *M* | 0.789 | 0.700 | **0.487** | **0.376** | **0.318** | **0.316** |
|  | LD.N^-^ |  | *ppc* | *rnpB* | *secA* | **16S** | ***rnpA*** | ***purC*** |
|  |  | *M* | 1.572 | 1.375 | 0.854 | 0.604 | 0.561 | 0.543 |
|  | LD^***^ |  | 16S | ***secA*** | ***purC*** | ***rnpA*** | ***rnpB*** | ***ppc*** |
|  |  | *M* | 1.377 | 1.292 | 1.188 | 1.148 | 1.045 | 1.024 |
| ***Nostoc* sp. PCC 7120** | CL.N^+^ |  | *rnpB* | *petB* | *ilvD* | *secA* | **16S** | ***rnpA*** |
|  |  | *M* | **0.475** | **0.377** | **0.282** | **0.212** | **0.200** | **0.193** |
|  | CL.N^-^ |  | 16S | *secA* | *rnpB* | ***petB*** | ***rnpA*** | ***ilvD*** |
|  |  | *M* | **0.437** | **0.433** | **0.377** | **0.350** | **0.346** | **0.343** |
|  | CL^***^ |  | *rnpA* | *petB* | ***ilvD*** | ***rnpB*** | **16S** | ***secA*** |
|  |  | *M* | 0.799 | 0.569 | 0.520 | **0.438** | **0.399** | **0.363** |
|  | LD.N^+^ |  | *petB* | *ilvD* | *rnpB* | ***secA*** | **16S** | ***rnpA*** |
|  |  | *M* | **0.434** | **0.380** | **0.318** | **0.285** | **0.264** | **0.226** |
|  | LD.N^-^ |  | *rnpA* | *rnpB* | **16S** | ***secA*** | ***ilvD*** | ***petB*** |
|  |  | *M* | 0.647 | **0.385** | **0.361** | **0.324** | **0.310** | **0.300** |
|  | LD^***^ |  | *rnpA* | *rnpB* | *ilvD* | ***petB*** | ***secA*** | **16S** |
|  |  | *M* | 0.661 | 0.552 | **0.439** | **0.427** | **0.415** | **0.395** |
| ***Synechocystis* sp. PCC 6803** | CL.N^+^ |  | *secA* | *ppc* | *rpoA* | 16S | ***rnpB*** | ***petB*** |
|  |  | *M* | 0.893 | 0.600 | 0.510 | **0.331** | **0.300** | **0.298** |
|  | LD.N^+^ |  | *rpoA* | *secA* | *ppc* | *rnpB* | ***petB*** | **16S** |
|  |  | *M* | 0.787 | 0.636 | 0.520 | **0.436** | **0.424** | **0.400** |

*CL – continuous light; LD – light/dark regimen; N^+^ – medium with combined nitrogen; N^-^ – medium without combined nitrogen.

**The best combination of genes obtained in the pair-wise analysis and the *M* values below the 0.5 threshold are in bold.

***Calculation performed pooling data from cells grown in both media and in the same light regimen.
