# Supplementary material for: Selection of Suitable Reference Genes for RT-qPCR Analyses in Cyanobacteria
Source: PLoS One. 2012 Apr 4;7(4):e34983. doi: 10.1371/journal.pone.0034983 (PMC3319621; doi:10.1371/journal.pone.0034983)
Supplement: Table S2 — Ranking of the candidate reference genes according to their stability value (M) calculated by NormFinder. (DOC) [file pone.0034983.s004.doc]

**Table S2.** Ranking of the candidate reference genes according to their stability value (*M*) calculated by NormFinder.

| **Organism** | **Condition^*^** |  | **Ranking (less stable to more stable)^**^** | | | | | |
| --- | --- | --- | --- | --- | --- | --- | --- | --- |
|  |  |  | 6 | 5 | 4 | 3 | 2 | 1 |
| ***Lyngbya aestuarii* CCY 9616** | CL.N^+^ |  | 16S | *rnpB* | *purC* | *rnpA* | *secA* | ppc |
|  |  | *M* | 1.797 | 0.662 | 0.249 | 0.131 | 0.068 | 0.068 |
|  | CL.N^-^ |  | *rnpB* | *ppc* | *purC* | 16S | *rnpA* | *secA* |
|  |  | *M* | 0.612 | 0.579 | 0.348 | 0.329 | 0.084 | 0.040 |
|  | CL^***^ |  | 16S | *rnpB* | *ppc* | ***purC*** | ***rnpA*** | *secA* |
|  |  | *M* | 1.812 | 1.417 | 0.744 | 0.300 | 0.197 | 0.181 |
|  | LD.N^+^ |  | *rnpB* | *purC* | *ppc* | *rnpA* | 16S | *secA* |
|  |  | *M* | 0.593 | 0.534 | 0.371 | 0.339 | 0.305 | 0.282 |
|  | LD.N^-^ |  | *secA* | *ppc* | *rnpB* | *purC* | *rnpA* | 16S |
|  |  | *M* | 1.805 | 1.501 | 0.979 | 0.232 | 0.128 | 0.128 |
|  | LD^***^ |  | 16S | *ppc* | *secA* | *rnpB* | ***purC*** | ***rnpA*** |
|  |  | *M* | 0.980 | 0.688 | 0.669 | 0.503 | 0.397 | 0.258 |
| ***Nostoc* sp. PCC 7120** | CL.N^+^ |  | *rnpB* | 16S | *petB* | *secA* | *ilvD* | *rnpA* |
|  |  | *M* | 0.325 | 0.207 | 0.195 | 0.179 | 0.170 | 0.166 |
|  | CL.N^-^ |  | *secA* | *ilvD* | *rnpA* | 16S | *petB* | *rnpB* |
|  |  | *M* | 0.116 | 0.079 | 0.068 | 0.062 | 0.044 | 0.027 |
|  | CL^***^ |  | *rnpA* | *petB* | *rnpB* | **16S** | ***secA*** | *ilvD* |
|  |  | *M* | 0.380 | 0.286 | 0.186 | 0.182 | 0.167 | 0.098 |
|  | LD.N^+^ |  | *petB* | *rnpB* | *ilvD* | *secA* | 16S | *rnpA* |
|  |  | *M* | 1.311 | 1.027 | 0.795 | 0.581 | 0.493 | 0.170 |
|  | LD.N^-^ |  | *rnpA* | *ilvD* | *petB* | 16S | *secA* | *rnpB* |
|  |  | *M* | 1.314 | 0.658 | 0.576 | 0.531 | 0.385 | 0.254 |
|  | LD^***^ |  | *rnpB* | *ilvD* | *petB* | ***rnpA*** | ***secA*** | 16S |
|  |  | *M* | 1.227 | 0.923 | 0.864 | 0.702 | 0.634 | 0.385 |
| ***Synechocystis* sp. PCC 6803** | CL.N^+^ |  | *rpoA* | *secA* | *petB* | *ppc* | *rnpB* | 16S |
|  |  | *M* | 1.866 | 1.317 | 0.812 | 0.274 | 0.244 | 0.084 |
|  | LD.N^+^ |  | 16S | *rpoA* | *secA* | *ppc* | *rnpB* | *petB* |
|  |  | *M* | 0.499 | 0.453 | 0.411 | 0.319 | 0.264 | 0.145 |

*CL – continuous light; LD – light/dark regimen; N^+^ – medium with combined nitrogen; N^-^ – medium without combined nitrogen.

**The best pair of genes comparing two different groups (N^+^ vs. N^-^) are in bold.

***Calculation performed pooling data from cells grown in both media and in the same light regimen.
