# Supplementary material for: Selection of Suitable Reference Genes for RT-qPCR Analyses in Cyanobacteria
Source: PLoS One. 2012 Apr 4;7(4):e34983. doi: 10.1371/journal.pone.0034983 (PMC3319621; doi:10.1371/journal.pone.0034983)
Supplement: Table S3 — Ranking of the candidate reference genes according to the Pearson's correlation coefficient (r) calculated against the BestKeeper index and probability values (p). (DOC) [file pone.0034983.s005.doc]

**Table S3.** Ranking of the candidate reference genes according to the Pearson’s correlation coefficient (r) calculated against the BestKeeper index and probability values (p).

| **Organism** | **Condition^*^** |  | **Ranking (less stable to more stable)^**^** | | | | | |
| --- | --- | --- | --- | --- | --- | --- | --- | --- |
|  |  |  | 6 | 5 | 4 | 3 | 2 | 1 |
| ***Lyngbya aestuarii* CCY 9616** | CL.N^+^ |  | *rnpB* | *secA* | *rnpA* | *purC* | 16S | *ppc* |
|  |  | *r* | 0.732 | 0.802 | 0.871 | 0.883 | 0.905 | 0.957 |
|  |  | *p*-Value | **0.007** | **0.002** | **0.001** | **0.001** | **0.001** | **0.001** |
|  | CL.N^-^ |  | *rnpB* | *purC* | *rnpA* | 16S | *secA* | *ppc* |
|  |  | *r* | 0.112 | 0.423 | 0.584 | 0.693 | 0.699 | 0.713 |
|  |  | *p*-Value | 0.726 | 0.170 | **0.047** | **0.012** | **0.011** | **0.009** |
|  | CL^***^ |  | *rnpB* | 16S | *purC* | *ppc* | *secA* | *rnpA* |
|  |  | *r* | 0.147 | 0.651 | 0.731 | 0.761 | 0.781 | 0.809 |
|  |  | *p*-Value | 0.491 | **0.001** | **0.001** | **0.001** | **0.001** | **0.001** |
|  | LD.N^+^ |  | *rnpA* | *purC* | *secA* | 16S | *rnpB* | *ppc* |
|  |  | *r* | 0.323 | 0.455 | 0.755 | 0.780 | 0.847 | 0.861 |
|  |  | *p*-Value | 0.306 | 0.136 | **0.005** | **0.003** | **0.001** | **0.001** |
|  | LD.N^-^ |  | *secA* | *rnpB* | *ppc* | *purC* | 16S | *rnpA* |
|  |  | *r* | 0.370 | 0.485 | 0.606 | 0.727 | 0.727 | 0.826 |
|  |  | *p*-Value | 0.236 | 0.111 | **0.037** | **0.007** | **0.007** | **0.001** |
|  | LD^***^ |  | *purC* | *secA* | *rnpB* | *rnpA* | *ppc* | 16S |
|  |  | *r* | 0.569 | 0.579 | 0.614 | 0.656 | 0.665 | 0.772 |
|  |  | *p*-Value | **0.004** | **0.003** | **0.001** | **0.001** | **0.001** | **0.001** |
| ***Nostoc* sp. PCC 7120** | CL.N^+^ |  | *ilvD* | *petB* | *rnpB* | *rnpA* |  | 16S*/secA* |
|  |  | *r* | 0.917 | 0.940 | 0.947 | 0.950 |  | 0.991 |
|  |  | *p*-Value | **0.001** | **0.001** | **0.001** | **0.001** |  | **0.001** |
|  | CL.N^-^ |  | *petB* | *secA* | *ilvD* | *rnpA* | *rnpB* | 16S |
|  |  | *r* | 0.198 | 0.684 | 0.797 | 0.813 | 0.846 | 0.942 |
|  |  | *p*-Value | 0.537 | **0.014** | **0.002** | **0.001** | **0.001** | **0.001** |
|  | CL^***^ |  | *petB* | *rnpA* | *ilvD* | *secA* | *rnpB* | 16S |
|  |  | *r* | 0.568 | 0.643 | 0.810 | 0.861 | 0.906 | 0.970 |
|  |  | *p*-Value | **0.004** | **0.001** | **0.001** | **0.001** | **0.001** | **0.001** |
|  | LD.N^+^ |  | *petB* | *ilvD* | *rnpB* | *rnpA* | 16S | *secA* |
|  |  | *r* | 0.491 | 0.547 | 0.548 | 0.653 | 0.936 | 0.951 |
|  |  | *p*-Value | 0.105 | 0.065 | 0.065 | **0.021** | **0.001** | **0.001** |
|  | LD.N^-^ |  | *rnpB* | *petB* | *rnpA* | *ilvD* | *secA* | 16S |
|  |  | *r* | 0.895 | 0.954 | 0.958 | 0.958 | 0.973 | 0.994 |
|  |  | *p*-Value | **0.001** | **0.001** | **0.001** | **0.001** | **0.001** | **0.001** |
|  | LD^***^ |  | *rnpB* | *petB* | *ilvD* | *rnpA* | *secA* | 16S |
|  |  | *r* | 0.850 | 0.865 | 0.890 | 0.927 | 0.941 | 0.985 |
|  |  | *p*-Value | **0.001** | **0.001** | **0.001** | **0.001** | **0.001** | **0.001** |
| ***Synechocystis* sp. PCC 6803** | CL.N^+^ |  | *secA* | *ppc* | *rpoA* | *petB* | *rnpB* | 16S |
|  |  | *r* | -0.356 | 0.702 | 0.894 | 0.952 | 0.987 | 0.989 |
|  |  | *p*-Value | 0.258 | 0.011 | 0.001 | 0.001 | 0.001 | 0.001 |
|  | LD.N^+^ |  | *secA* | *rpoA* | *ppc* | *petB* | 16S | *rnpB* |
|  |  | *r* | 0.149 | 0.192 | 0.725 | 0.899 | 0.923 | 0.935 |
|  |  | *p*-Value | 0.642 | 0.549 | **0.008** | **0.001** | **0.001** | **0.001** |

*CL – continuous light; LD – light/dark regimen; N^+^ – medium with combined nitrogen; N^-^ – medium without combined nitrogen.

**Significant *p*-Values are in bold.

***Calculation performed pooling data from cells grown in both media and in the same light regimen.
