# Supplementary material for: Selection of Suitable Reference Genes for RT-qPCR Analyses in Cyanobacteria
Source: PLoS One. 2012 Apr 4;7(4):e34983. doi: 10.1371/journal.pone.0034983 (PMC3319621; doi:10.1371/journal.pone.0034983)
Supplement: Table S4 — Candidate reference genes accession number and location. (DOC) [file pone.0034983.s006.doc]

**Table S4.** Candidate reference genes accession number and location.

| **Organism** | **Genes** | **Accession number**  **(nucleotides, locus tag)** |
| --- | --- | --- |
| ***Lyngbya aestuarii* CCY 9616** | *ilvD* | NZ_AAVU01000015.1 (64391 to 66073^*^, L8106_26847) |
|  | *mrp* | NZ_AAVU01000008.1 (141808 to 142878, L8106_17697) |
|  | *petB* | NZ_AAVU01000005.1 (107734 to 108402^*^, L8106_09696) |
|  | *ppc* | NZ_AAVU01000013.1 (80928 to 84044, L8106_19036) |
|  | *prsA* | NZ_AAVU01000001.1 (11840 to12856^*^, L8106_04291) |
|  | *purC* | NZ_AAVU01000001.1 (122582 to 123316, L8106_04766) |
|  | *rnpA* | NZ_AAVU01000059.1 (974 to 1381, L8106_29250) |
|  | *rnpB* | NZ_AAVU01000017.1 (76166 to 76621^*^, N/A) |
|  | *rpoA* | NZ_AAVU01000003.1 (146690 to 147634^*^, L8106_15090) |
|  | *rps1B* | NZ_AAVU01000005.1 (123246 to 124133^*^, L8106_09766) |
|  | *rrn16Sa* | NZ_AAVU01000008.1 (780 to 2269^*^, N/A) |
|  | *rrn16Sb* | NZ_AAVU01000018.1 (81295 to 82783, N/A) |
|  | *secA* | NZ_AAVU01000009.1 (97343 to 100138^*^, L8106_16729) |
| ***Nostoc* sp. PCC 7120** | *ilvD* | NC_003272.1 (3367200 to 3368891, alr2771) |
|  | *mrp* | NC_003272.1 (754691 to 755761,alr0652) |
|  | *petB* | NC_003272.1 (4130387 to 4131034, alr3421) |
|  | *ppc* | NC_003272.1 (5788301 to 5791249^*^,all4861) |
|  | *prsA* | NC_003272.1 (5570574 to 5571566,alr4670) |
|  | *purC* | NC_003272.1 (2730420 to 2731157,alr2268) |
|  | *rnpA* | NC_003272.1 (4122806 to 4123228, alr3413) |
|  | *rnpB* | NC_003272.1 (4950355 to 4950814^*^, allrs04) |
|  | *rpoA* | NC_003272.1 (5038362 to 5039309^*^,all4191) |
|  | *rps1B* | NC_003272.1 (1261663 to 1262580,alr1078) |
|  | *rrn16Sa* | NC_003272.1 (2375734 to 2377222, allrr01) |
|  | *rrn16Sb* | NC_003272.1 (2500525 to 2502013, allrr04) |
|  | *rrn16Sc* | NC_003272.1 (4918283 to 4919771^*^, allrr09) |
|  | *rrn16Sd* | NC_003272.1 (5945700 to 5947188^*^, allrr12) |
|  | *secA* | NC_003272.1 (5778711 to 5781503, alr4851) |
| ***Synechocystis* sp. PCC 6803** | *ilvD* | NC_000911.1 (3498903 to 3500588, slr0452) |
|  | *mrp* | NC_000911.1 (2596787 to 2597848, slr0067) |
|  | *petB* | NC_000911.1 (2428010 to 2428678, slr0342) |
|  | *ppc* | NC_000911.1 (1994147 to 1997251^*^, sll0920) |
|  | *prsA* | NC_000911.1 (2938630 to 2939631^*^, sll0469) |
|  | *purC* | NC_000911.1 (1030195 to 1030989, slr1226) |
|  | *rnpA* | NC_000911.1 (1826911 to 1827285, slr1469) |
|  | *rnpB* | NC_000911.1 (153057 to 153655, 6803s01) |
|  | *rpoA* | NC_000911.1 (829141 to 830085^*^, sll1818) |
|  | *rps1B* | NC_000911.1 (1815454 to 1816371, slr1984) |
|  | *rrn16Sa* | NC_000911.1 (2452187 to 2453675^*^, 6803r03) |
|  | *rrn16Sb* | NC_000911.1 (3325053 to 3326541, 6803r04) |
|  | *secA* | NC_000911.1 (2647386 to 2650184^*^, sll0616) |

*Complement sequence.

N/A – not-available.
