# Supplementary material for: Selection of Suitable Reference Genes for RT-qPCR Analyses in Cyanobacteria
Source: PLoS One. 2012 Apr 4;7(4):e34983. doi: 10.1371/journal.pone.0034983 (PMC3319621; doi:10.1371/journal.pone.0034983)
Supplement: Table S5 — Primer nucleotide sequences of genes tested only in preliminary RT-qPCR analysis. (DOC) [file pone.0034983.s007.doc]

**Table S5.** Primer nucleotide sequences of genes tested only in preliminary RT-qPCR analysis.

| **Organism** | **Genes** | **Primer name** | **Primer Sequence (5’ → 3’)** | **T_a_ (ºC)** |
| --- | --- | --- | --- | --- |
| ***Lyngbya aestuarii* CCY 9616** | *ilvD* | ilvDF | GGCTGTGATAAGAATATGC | 51 |
|  |  | ilvDR | CCACCGTAAACAAAGATAG |  |
|  | *mrp* | mrpF | GAACTGAATATGATTCGCAACG | 51 |
|  |  | LmrpR | ACAGGCGGGAGTGGTGAG |  |
|  | *petB* | LpetB1F | GTATGATGGTATTGATGATGATTC | 51 |
|  |  | LpetB1R | AGGTATGTAAGGTGTAGAAGC |  |
|  | *prsA* | prsAF | TTAAATGTGATTGGTGATGT | 56 |
|  |  | LprsAR | AGTCGCACAAGCATAAAC |  |
|  | *rpoA* | LrpoAF | ATATCTGGACGAATGGTA | 51 |
|  |  | LrpoAR | CGTTTGAGACAGTTATAGG |  |
|  | *rps1B* | Lrps1F | TTGAATATACGAGCGATGG | 56 |
|  |  | Lrps1R | TGAGAGGAGAACTTGACC |  |
| ***Nostoc* sp. PCC 7120** | *mrp* | mrpF | GAACTGAATATGATTCGCAACG | 51 |
|  |  | NmrpR | TGGCAATCTTCAACAATAAATTCC |  |
|  | *ppc* | ppcF | TATCGCCAAGAACCCTATCG | 51 |
|  |  | NppcR | CCTGTTTCCGTCAAGTTATGC |  |
|  | *prsA* | prsAF | TTAAATGTGATTGGTGATGT | 56 |
|  |  | NprsAR | ATCGTTCAACAGCAGGAG |  |
|  | *purC* | purCF | GATACCTGTCGTTTGTGG | 51 |
|  |  | NpurCR | CTCCTGATAAGCATCTTCTAC |  |
|  | *rpoA* | NrpoAF | ACTCCGACATTCCAGACG | 51 |
|  |  | rpoAR1 | CGCTTGAGACAGTTATAGGC |  |
|  | *rps1B* | Nrps1F | GGTGGATGTGAAGGTGAC | 51 |
|  |  | Nrps1R | CAGCCGTGAGAGTATGAC |  |
| ***Synechocystis* sp. PCC 6803** | *ilvD* | ilvDF2 | ATGGGAACCGAGGGGATG | 51 |
|  |  | LilvDR | TTATCACAACCGCCAATCG |  |
|  | *mrp* | SmrpF | GGCATTATCCGTCAGTTCC | 56 |
|  |  | SmrpR | GGCACCGATTGAGTTAGG |  |
|  | *prsA* | SprsAF | CGTCGTCAGAGCCATAAC | 51 |
|  |  | SprsAR | GAGCAAACGGGAGCCTTC |  |
|  | *purC* | purCF | GATACCTGTCGTTTGTGG | 56 |
|  |  | SpurCR | TTCCACTGTTTGATAAGCG |  |
|  | *rnpA* | rnpAF1 | AACGTCAAATTCGAGCCG | 56 |
|  |  | rnpAR1 | AACAACTGCTCTAATTCTTGC |  |
|  | *rps1B* | Srps1F | TTGGCAGAGTTGGAGGAG | 56 |
|  |  | Srps1R | CGAGGATATGGGCTTTGAG |  |
